# Unilateral biportal endoscopic discectomy versus percutaneous endoscopic lumbar discectomy in the treatment of lumbar disc herniation: a retrospective study

**DOI:** 10.1186/s13018-022-02929-5

**Published:** 2022-01-15

**Authors:** Hao-Wei Jiang, Cheng-Dong Chen, Bi-Shui Zhan, Yong-Li Wang, Pan Tang, Xue-Sheng Jiang

**Affiliations:** Department of Orthopaedics, Huzhou Central Hospital, No.1558, Sanhuan North Road, Wuxing District, Huzhou, 313000 Zhejiang Province China

**Keywords:** Lumbar disc herniation, Percutaneous endoscopic lumbar discectomy, Unilateral biportal endoscopic discectomy, Hidden blood loss

## Abstract

**Background:**

Unilateral biportal endoscopic discectomy (UBE) is a rapidly growing surgical method that uses arthroscopic system for treatment of lumbar disc herniation (LDH), while percutaneous endoscopic lumbar discectomy (PELD) has been standardized as a representative minimally invasive spine surgical technique for LDH. The purpose of this study was to compare the clinical outcomes between UBE and PELD for treatment of patients with LDH.

**Methods:**

The subjects consisted of 54 patients who underwent UBE (24 cases) and PELD (30 cases) who were followed up for at least 6 months. All patients had lumber disc herniation for 1 level. Outcomes of the patients were assessed with operation time, incision length, hospital stay, total blood loss (TBL), intraoperative blood loss (IBL), hidden blood loss (HBL), complications, total hospitalization costs, visual analogue scale (VAS) for back and leg pain, the Oswestry disability index (ODI) and modified MacNab criteria.

**Results:**

The VAS scores and ODI decreased significantly in two groups after operation. Preoperative and 1 day, 1 month, 6 months after operation VAS and ODI scores were not significantly different between the two groups. Compared with PELD group, UBE group was associated with higher TBL, higher IBL, higher HBL, longer operation time, longer hospital stay, longer incision length, and more total hospitalization costs. However, a dural tear occurred in one patient of the UBE group. There was no significant difference in the rate of complications between the two groups.

**Conclusions:**

Application of UBE for treatment of lumbar disc herniation yielded similar clinical outcomes to PELD, including pain control and patient satisfaction. However, UBE was associated with various disadvantages relative to PELD, including increased total, intraoperative and hidden blood loss, longer operation times, longer hospital stays, and more total hospitalization costs.

## Background

Lumbar disc herniation (LDH) is a primary cause of back pain and sciatica, affecting 1% to 5% of the population annually [1]. Although nonsurgical care remains the mainstay of initial treatment, discectomy surgery is applied to effectively alleviate symptoms that persist for prolonged periods of time [2]. Minimally invasive spine surgery technology has been widely used for treatment of LDH [3]. In percutaneous endoscopic lumbar discectomy (PELD), which was first described in the early 1980s [4], a surgeon uses a unilateral single channel endoscopy to directly reach the target position through Kambin’s triangle, remove the herniated disc tissue and release nerve roots [5]. PELD has not only yielded successful outcomes compared to conventional open or microendoscopic surgery, but has shown advantages in controlling muscular trauma, shortening hospital stay, and maintaining the spinal segment stability [6]. Consequently, it has been standardized as a representative minimally invasive spine surgical technique for LDH [7].

Unilateral biportal endoscopic discectomy (UBE) is a rapidly growing surgical method that uses arthroscopic system for treatment of LDH. Notably, the technique has two independent channel endoscopies, which provide a clear and magnified surgical field that improves operational flexibility, helps the surgeon to perform precise and extensive decompression [8]. Results from preliminary clinical trials, and the complications of UBE in LDH treatment have been documented [9]. In the present study, we compared the safety and efficacy between UBE and PELD for treatment of patients with LDH.

## Material and methods

### Patients

Form June 2020 to January 2021, the data of the consecutive hospitalized patients with LDH undergoing UBE or PELD in Huzhou Central Hospital were retrospectively collected. According to the following inclusion and exclusion criteria. 54 patients were suitable for our study.

The inclusion criteria included the following: (1) clinical symptoms of back or radiating pain; (2) magnetic resonance images with single level herniation associated with symptoms; (3) conservative treatment failed over three months; (4) follow-up of at least 6 months.

The exclusion criteria included the following: (1) segmental instability; (2) recurrent LDHs; (3) severe central or lateral-recess stenosis; (4) Cauda equina syndrome. (5) spinal tumors; (6) ankylosing spondylitis; (7) lumbar vertebral fracture.

### Surgery

All surgical procedures were performed by one experienced surgeon.

#### UBE

**Fig. 1:**
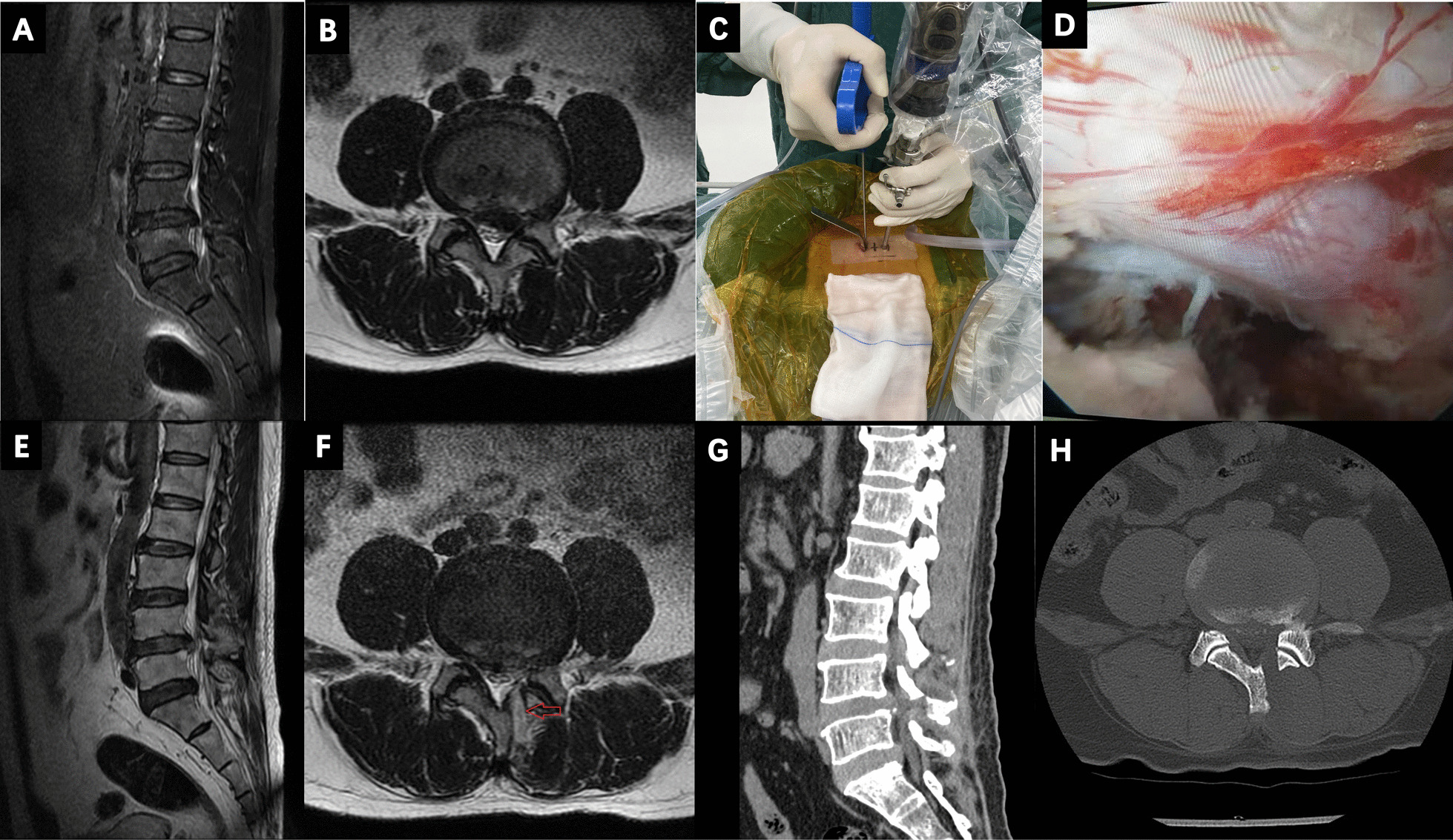
**A**, **B** Preoperative sagittal and axial magnetic resonance images in a 34-year-old male patient complaining of left radicular leg pain, showing L4–L5 disc herniation on the left side. **C** Image of the patient who underwent unilateral biportal endoscopic discectomy. **D** Endoscopic image showing the relaxation of L5 nerve root after decompression. **E**, **F** Postoperative sagittal and axial magnetic resonance images made one day after surgery. High signal intensity (red arrow) indicating blood extravasates. **G**, **H** Postoperative computed tomography made two days after surgery showing defect of lamina after partial laminotomy

All surgical operations were performed under general anesthesia. Patients were placed in a prone position on a radiolucent table. The surgeon confirmed the target intervertebral site in a frontal view and stood on the left side of the patient. Two skin insertion points were made at 1–1.5 cm lateral to the midline, and the surface of the inferior margin of the upper lamina was the incision for endoscope insertion, while the surface of superior margin of the lower lamina was the incision for surgical instruments insertion. The soft tissue was endoscopically cauterized with radiofrequency ablation, to create working space. Next, the spinolaminar junction at the target intervertebral site were identified, a partial laminotomy was performed, part of the inferior lamina of the upper lumbar spine and superior lamina of the lower lumbar spine were removed using an electric drill. The interlaminar ligament was dissected and removed using Kerrison punch and a radiofrequency probe, followed by dissection and exposure of the annulus of the protruding intervertebral disc. Prior to discectomy, overgrown epidural vessels were coagulated carefully to minimize bleeding. The ruptured fragments were then removed using Kerrison punches and pituitary forceps. Finally, decompression of the nerve root and pulsation of the dura mater was confirmed, a drain inserted, and the surgical incision closed (Fig. 1).

#### PELD

**Fig. 2:**
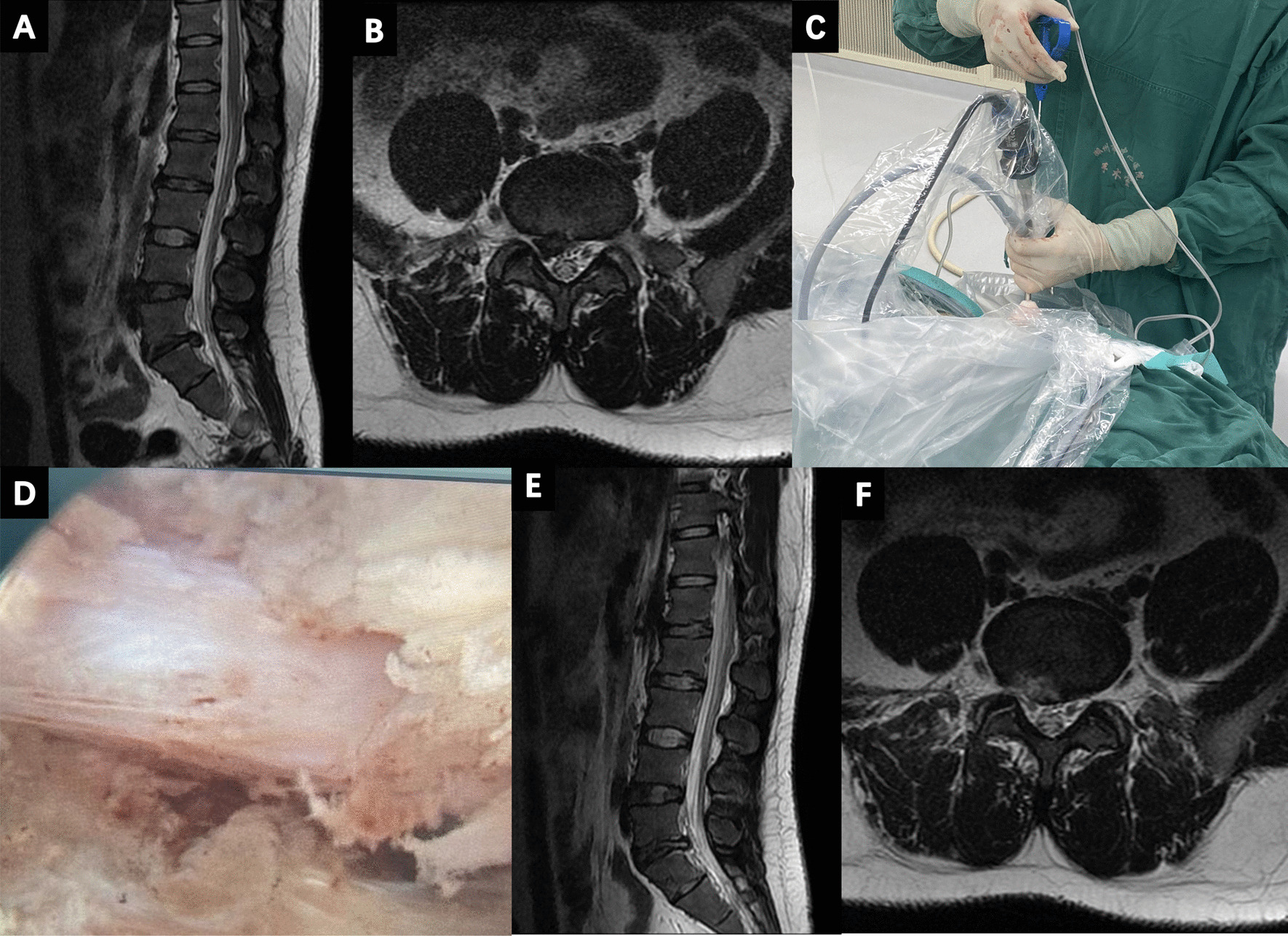
**A**, **B** Preoperative sagittal and axial magnetic resonance images in a 29-year-old male patient complaining of right radicular leg pain, showing L5-S1 disc herniation on the right side. **C** Image of the patient who underwent percutaneous endoscopic lumbar discectomy. **D** Endoscopic image showing the relaxation of S1 nerve root after decompression. **E**, **F** Postoperative sagittal and axial magnetic resonance images made one day after surgery

All surgical operations were performed under local anesthesia. Patients were lying lateral with the affected side facing upwards. Under X-ray guidance, the puncture site was confirmed and marked by the surgeon, an 18-gauge spinal needle was then inserted into the target intervertebral disc level. In the lateral view, the needle tip was located at the posterior vertebral bodyline. In the anterio-posterior view, the same needle tip layed at the medial pedicular line. A guidewire was inserted through the spinal needle, and then the needle was removed. Next, an incision was made in order to insert a tapered cannulated obturator along the guidewire, followed by insertion of the obturator into the disc with hammering. Thereafter, a bevel-ended, oval-shaped working cannula was inserted. Finally, an endoscope was inserted through the working cannula, and the herniated disc removed with endoscopic forceps and radiofrequency probe, and the surgical incision closed (Fig. 2).

### Data collection

General information included age, gender, height and weight for body-mass index (BMI) calculation, follow-up duration, disc location and disc level. Perioperative data included hematocrit (Hct), Hemoglobin (Hb), total blood loss (TBL), intraoperative blood loss (IBL), hidden blood loss (HBL), operation time, incision length, hospital stay, complications and total hospitalization costs were collected and evaluated.

The TBL was calculated according to the formula proposed by Gross [10].

$$\mathrm{TBL }(\mathrm{mL})=\mathrm{EBV}(\mathrm{L})\times \frac{{\mathrm{Hct}}_{\mathrm{pre}}-{\mathrm{Hct}}_{\mathrm{post}}}{{\mathrm{Hct}}_{\mathrm{ave}}}\times 1000$$

where EBV = patient’s estimated blood volume; ${\mathrm{Hct}}_{\mathrm{pre}}$= patient’s pre-operative hematocrit; ${\mathrm{Hct}}_{\mathrm{post}}$ = patient’s post-operative hematocrit;${\mathrm{Hct}}_{\mathrm{ave}}$= (${\mathrm{Hct}}_{\mathrm{pre}}-{\mathrm{Hct}}_{\mathrm{post}}$)/2; and EBV was calculated on the basis of the Nadler formula [11].

$${\text{EBV }}\left( {{\text{ml}}} \right) = {\text{k1}} \times {\text{height}}\left( {\text{m}} \right) \times {3} + {\text{k2}} \times {\text{weight }}\left( {{\text{kg}}} \right) + {\text{k3}},$$

For men, k1 = 0.3669, k2 = 0.03219, and k3 = 0.6041, for women k1 = 0.3561, k2 = 0.03308, and k3 = 0.1833.

The IBL was calculated as the suction fluid minus the liquid used to irrigation during the surgery, and the weight difference between gauzes and surgical towels before and after surgery.

The HBL was calculated as follows: HBL = TBL—IBL—postoperative drainage. In UBE group, the postoperative drainage was recorded as the amount of blood in the drainage bag until it was removed when the drainage flow was < 50 ml/day. In PELD group, no drains were placed, and the postoperative drainage was calculated as zero.

The clinical outcomes were evaluated by collecting questionnaire answers [visual analogue scale (VAS) for measuring back and leg pain intensity and Oswestry disability index (ODI) for disability] preoperatively and 1 day, 1 month and 6 months postoperatively. The patient satisfaction rate of clinical outcomes was assessed by modified MacNab criteria, which include four grades: excellent, good, fair, and poor, excellent and good were recognized as clinically satisfactory.

### Statistical analysis

All statistical analyses were performed using SPSS software. (Version 26.0, Chicago, IL, USA). Intergroup comparisons were employed using independent samples t-test, Chi-Square tests and Mann–Whitney U tests; Intragroup comparisons were conducted using paired t test. Comparisons with values of *P* < 0.05 were considered statistically significant.

## Results

### General information

A total of 54 patients in UBE (24 cases) and PELD (30 cases) met the inclusion criteria, No significant differences were observed between both groups in preoperative demographics and clinical characteristics (*P* > 0.05) (Table 1).

**Table 1:** The general information of UBE and PELD

	PELD (*n* = 30)	UBE (*n* = 24)	*p* value
Age (years)	46.10 ± 10.45	46.25 ± 12.78	0.963
*Gender*			
M	13	10	0.902
F	17	14	
BMI (kg/m^2^)	22.09 ± 2.90	22.19 ± 3.46	0.905
Follow-up duration (months)	6.40 ± 0.29	6.36 ± 0.21	0.599
*Disc location*			
Central	7	6	0.887
Paracentral	23	18	
*Disc level*			
L3-4	3	1	0.606
L4-5	18	15	
L5-S1	9	8	

### Perioperative outcomes and complications

Compared with PELD group, UBE group was associated with more Hb loss, more Hct loss, higher TBL, higher IBL, higher HBL, longer operation time, longer hospital stay, longer incision length, and more total hospitalization costs (*P* < 0.05) (Table 2). One dural tear occurred in UBE group during disc resection. and this patient was observed for 24 h after surgery with absolute bed rest and adequate fluid infusion. No complications occurred in PELD group (*P* > 0.05) (Table 2).

**Table 2:** Comparison of perioperative date of UBE and PELD

	PELD (*n* = 30)	UBE (*n* = 24)	*p* value
Pre-op Hb (g/L)	128.90 ± 10.36	127.29 ± 10.72	0.579
Post-op Hb (g/L)	124.23 ± 10.30	117.46 ± 10.44	0.021
Hb loss, g/L (g/L)	4.67 ± 5.16	9.83 ± 8.17	0.007
Pre-op Hct (%)	38.62 ± 2.08	38.13 ± 2.22	0.407
Post-op Hct (%)	38.20 ± 2.02	35.12 ± 2.54	< 0.001
Hct loss (%)	0.43 ± 0.26	3.01 ± 1.41	< 0.001
Total blood loss (ml)	43.68 ± 24.54	332.10 ± 190.17	< 0.001
Intraoperative blood loss (ml)	13.04 ± 6.22	92.16 ± 43.57	< 0.001
Hidden blood loss (ml)	30.64 ± 22.29	195.62 ± 130.44	< 0.001
Operation time (min)	64.63 ± 13.13	117.54 ± 20.36	< 0.001
Hospital stay (days)	3.33 ± 0.80	7.04 ± 1.55	< 0.001
Incision length (mm)	10.31 ± 1.22	21.33 ± 1.55	< 0.001
Complications	0	1	0.259
Total hospitalization costs (¥)	20,341.35 ± 1062.41	25,068.77 ± 1177.22	< 0.001

### Clinical outcomes

Postoperative VAS scores and ODI decreased significantly in the two groups compared with preoperative scores (*P* < 0.05) (Table 3). No significant differences existed in VAS and ODI scores between the two groups preoperatively, 1 day, 1 month, and 6 months after operation (*P* > 0.05) (Table 3). According to MacNab criteria, patient satisfaction rates were 83.33% and 86.67% in UBE group and PELD group, There was no significant difference was observed in patient satisfaction rates between both groups (*P* > 0.05) (Table 3).

**Table 3:** Clinical outcomes of UBE and PELD

	PELD (*n* = 30)	UBE (*n* = 24)	*p* value
*VAS back*			
Preoperative	6.00 ± 0.95	5.75 ± 0.99	0.349
1 day after operation	1.87 ± 1.33	1.54 ± 1.18	0.353
1 month after operation	0.93 ± 1.41	0.79 ± 1.06	0.685
6 months after operation	0.47 ± 0.78	0.46 ± 0.66	0.967
*VAS leg*			
Preoperative	7.10 ± 1.56	7.04 ± 2.12	0.908
1 day after operation	2.20 ± 1.94	1.96 ± 1.52	0.619
1 month after operation	1.37 ± 1.33	0.92 ± 1.53	0.354
6 months after operation	0.53 ± 0.78	0.67 ± 1.34	0.649
*ODI*			
Preoperative	64.20 ± 9.46	62.25 ± 13.57	0.537
1 day after operation	21.67 ± 18.35	21.00 ± 13.12	0.881
1 month after operation	13.80 ± 21.40	11.42 ± 16.00	0.650
6 months after operation	8.00 ± 14.77	6.50 ± 9.08	0.665
Modified MacNab evaluation (excellent/good/fair/poor)	17/9/3/1	13/7/4/0	
Excellent/good rate	86.67%	83.33%	0.732

## Discussion

The significant improvements in pain score and functional status observed in both groups at 1 day, 1 month, and 6 month follow-ups were consistent with prior finding [12–14]. The modified MacNab criteria revealed an acceptable patient satisfaction in UBE and PELD groups, indicating that both techniques were equally effective in treating LDH. However, UBE technique was linked to significantly higher TBL, higher IBL, higher HBL, longer operation times, longer hospital stays, longer incision lengths, and more total hospitalization costs.

The rapid development of high-resolution endoscopes has increased interest in minimally invasive spine surgery technology. with more emphasis now on enhanced recovery after surgery, PELD, which results in favorable long-term outcomes [15], has been standardized as a representative minimally invasive spine surgical technique for LDH treatment [7]. The integrity of the paraspinal muscle was preserved by directly reaching the target position using a muscle-splitting technique with sequential dilators and blunt obturator [12]. Notably, PELD has various advantages over other minimally invasive discectomy approaches, such as MD and MED, including less occurrence of paravertebral muscle injury, preservation of bony structures, and rapid recovery [15, 16]. Use of the UBE technique for treatment of disc herniation was first described by Antoni in 1966 [17]. Since then, the technique has been rapidly applied in recent years. Generally, it combines muscle splitting and small extent muscle-stripping techniques to create a working space in the interlaminar space using serial dilators, bipolar radiofrequency probe, and continuous saline irrigation. Consequently, it provides a clear visualization of neural elements, surrounding soft tissues, vascular and bony structures [8], thereby creating a conducive environment for delicate nerve manipulation process and an easy and safe decompression. The UBE technique provides a minimally invasive option for nerve decompression in patients with posterior epidural migration of herniation disc, lumbar spinal stenosis and lumbar osteoporotic vertebral compression fracture [18–20].

However, the learning curve for UBE technique remains steep. Creating the working space and identifying spinolaminar junction were a great challenge in our first several cases; however, our personal experience indicates that, a definite preoperative orientation and trajectory, a clear anatomic knowledge and familiarity with UBE instruments are correlated with a shorter procedure duration. Furthermore, adequate hemostasis and delicate dissection of meningo-vertebral ligament were vital to avoid complications such as such as postoperative epidural hematoma and iatrogenic durotomy.

To our knowledge, only one report has compared median follow-up clinical outcomes between UBE and PELD for LDH treatment [21]. Specifically, 40 LDH patients in PELD (20 cases) and UBE (20 cases) group were analyzed, and favorable outcomes were achieved over a 6-month follow-up period. This study included patients with single L4/5 LDH and collected and evaluated their intraoperative hemorrhages. The present study reviewed patients with L3-4, L4-5 and L5-S1 LDH and then analyzed intraoperative, hidden and total blood loss as a parameter for understanding perioperative blood loss.

In spinal surgery, total blood loss is composed of visible blood loss, including intraoperative hemorrhages and drainage, as well as HBL in which blood extravasates into tissues and accumulates in the surgical field. Notably, compared with visible blood loss, HBL is often overlooked by spine surgeons [22], while an association is found between HBL and perioperative complications [23, 24]. Zhang et al. [25] found that HBL is seriously underestimated and accounts for a large percentage of TBL in minimally invasive transforaminal lumbar interbody fusion. On the other hand, Ao et al. [26] demonstrated that a large amount of HBL existed during percutaneous endoscopic surgery. In this study, PELD group exhibited significantly lower HBL than UBE group, mainly because PELD utilizes an existing natural access, intervertebral foramina, and allowed the surgeon to directly reach the disc without excision of the lamina, and ligamentum flavum. However, the partial laminotomy employed in UBE technique may have caused bleeding of cancellous bone. Our findings were consistent with those of Wang et al. [27] who reported a 469.5 mL of HBL following UBE surgery. However, their study included patients with lumbar degenerative diseases, including LDH, lumbar stenosis, and spondylolisthesis. This study only recruited LDH patients. Surgeons need to pay more attention to HBL within the perioperative management of UBE surgery to further improve patient safety and postoperative outcomes.

Regarding complications, a dural tear occurred in one patient of the UBE group. According to previous research, a dural tear is the most prevalent UBE complication, as evidenced by an incidence rate ranging from 1.5 to 5.8% [9]. The risk factors for dural tear include damage to the dura mater by instruments or radiofrequency, adhesion in the spinal canal, giant disc fragments, and loosened dura [28]. Therefore, it is particularly important to delicately dissect the meningo-vertebral ligament. For small tears of less than 4 mm, absolute bed rest and simple observation are recommended [29], while for larger defects of more than 10 mm, open repair is considered a safe treatment method [30]. Nevertheless, the endoscopic dural repair technique requires further development to improve efficacy and patient safety [31]. We found no dural tear in PELD group. Chen et al. [7] recently evaluated variability in tissue pain during PELD for LDH treatment, and found that with regards to VAS leg, the most intense pain originated from the nerve root/dural sac. This could help surgeons avoid nerve injury, dural sac tear, and other surgical complications by preliminarily judging the nature of tissues according to intraoperative VAS scores of patients under local anesthesia.

This study had several limitations. Firstly, there were no radiologic outcomes such as stability of lumbar spine and adjacent segment degeneration. Secondly, the study adopted a retrospective design. Future studies are expected to adopt prospective, multicenter studies with larger samples sizes, and also compare uniportal and other microscopic endoscopic procedures with long-term clinical outcomes.

## Conclusion

Application of UBE for treatment of lumbar disc herniation yielded similar clinical outcomes to PELD, including pain control and patient satisfaction. However, UBE was associated with various disadvantages relative to PELD, including increased total, intraoperative and hidden blood loss, longer operation times, longer hospital stays, and more total hospitalization costs. Although UBE is an effective and minimally invasive surgical technique for treatment of LDH patients, further improvements are needed to narrow down these differences compared to PELD.

## Data Availability

The datasets used and analysed during the current study are available from the corresponding author on reasonable request.

## Abbreviations

LDH: Lumbar disc herniation
PELD: Percutaneous endoscopic lumbar discectomy
UBE: Unilateral biportal endoscopic discectomy
BMI: Body-mass index
Hct: Hematocrit
Hb: Hemoglobin
TBL: Total blood loss
IBL: Intraoperative blood loss
HBL: Hidden blood loss
EBV: Estimated blood volume
VAS: Visual analogue scale
ODI: Oswestry disability index for disability
